# Segmental dyskeratosis congenita – A diagnostic challenge^؆^

**DOI:** 10.1016/j.abd.2025.501226

**Published:** 2025-11-04

**Authors:** Hiram Larangeira de Almeida Junior, Cecília Mazzoleni Facchini, Camila Urach Colpo, Manuela Sallaberry Maciel, Ana Júlia Baschirotto Custódio, Aline Paganelli

**Affiliations:** a Postgraduate Degree in Health and Behavior, Universidade Católica de Pelotas, Pelotas, RS, Brazil; b Dermatology Service, Santa Casa de Porto Alegre, Porto Alegre, RS, Brazil; c Faculdade de Medicina, Universidade Católica de Pelotas Pelotas, RS, Brazil; d Centro de Anatomia Patológica, Pelotas, RS, Brazil

Dear Editor,

Dyskeratosis congenita (DC) is a telomeropathy with a classic triad: dystrophic nails, oral leukoplakia, and reticular cutaneous pigmentation.^1^, ^2^, ^3^ It occurs primarily in males and can be autosomal recessive or dominant, or an X-linked recessive disorder. A mutation in the *DKC1* (Diskerin 1) gene on the X chromosome was initially discovered, resulting in reduced telomerase activity and consequent telomere shortening, marking the first link between telomeres and disease in humans.^1^

Mutations in the *ACD*, *CTC1*, *NAF1*, *NHP2*, *NOP10*, *PARN*, *POT1*, *RTEL1*, *STN1*, *TERC*, *TERT*, *TINF2*, *WRAP53*, and *ZCHC8* genes have also been described,^2^ increasing the complexity of the disease. The *TINF2* (12.5%), *RTEL1* (14.5% ‒ AD and 7.5% ‒ AR), *TERT* (23.5%), and *DKC1* (16% both gender and 22% in males) genes are the most commonly affected.^1^

A 40-year-old Caucasian female patient with palmoplantar and nail alterations, as well as asymptomatic patches on the trunk, which had begun at the age of four, was examined.

On examination, the entire right sole, left heel, and focally on the right palm, revealed hyperkeratosis with lamellar desquamation (Fig. 1A-B). Nail involvement was heterogeneous: micronychia was observed on the right from the second to the fourth toes (Fig. 1C), where the plantar region was most affected. Anonychia also appeared on the right second and fourth fingers. The left hand showed a decrease in nail plate width of the third finger, pterygium of the second, and canalicular dystrophy of the fourth finger (Fig. 2).

**Fig. 1:**
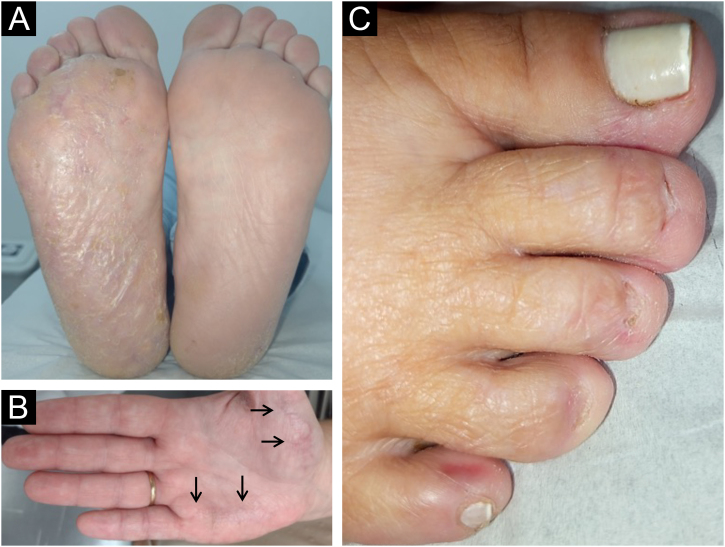
(A) Diffuse hyperkeratosis on the right sole. (B) Focal hyperkeratosis on the right palm (arrows). (C) Micronychia of the second to fourth right toes.

**Fig. 2:**
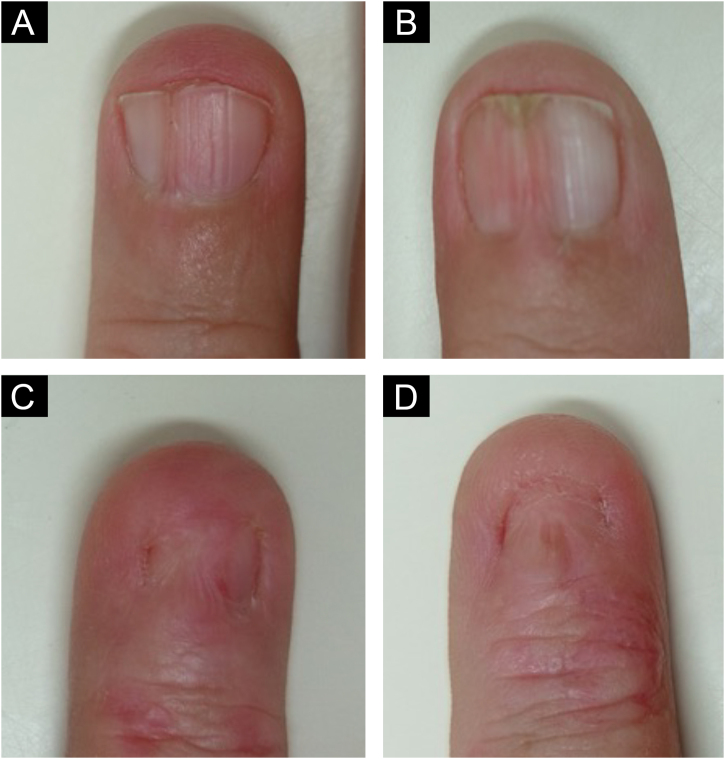
Variability of nail involvement (A) Vertical ridges. (B) Mild pterygium. (C) Severe pterygium. (D) Anonychia. The skin of the distal phalanges of the most affected nails (C‒D) shows mild erythema and desquamation.

The patient also had irregular, reticulated hyperchromic patches, bordering the midline on the abdomen (Fig. 3A). Segmental pigmentary alterations with hyper- and hypochromia were also observed on the dorsum of the right hand (Fig. 3B). The oral mucosa showed no abnormalities.

**Fig. 3:**
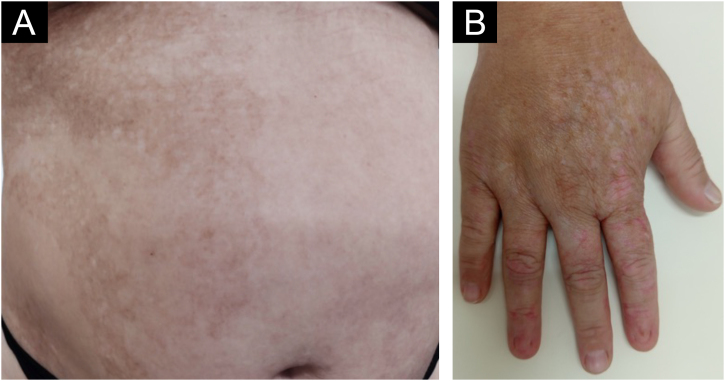
(A) Segmental hyperpigmented macules on the right abdomen. (B) Segmental hypo- and hyperpigmentation located on the dorsum of the right hand.

Laboratory tests were unremarkable; mycological examination of the soles was negative. Anatomopathological analysis of the hyperkeratotic lesion on the right palm showed significant rectification of the epidermis with orthohyperkeratosis. Fibrosis and proliferation of small vessels were observed in the dermis (Fig. 4A). The hyperpigmentation on the abdomen revealed vacuolar degeneration foci of basal cells with mild pigment incontinence (Fig. 4B). Fontana-Masson staining of the hyperpigmented area demonstrated irregular distribution of melanin in the epidermis and dermal melanin (Fig. 4C).

**Fig. 4:**
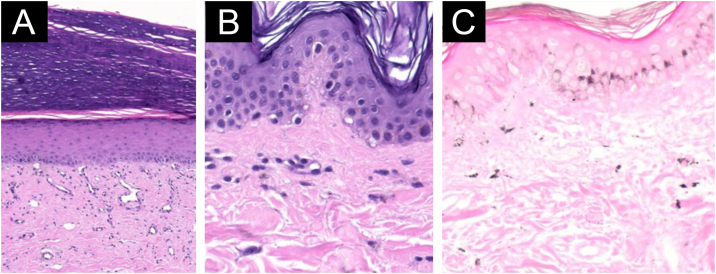
Light microscopy showing nonspecific findings. (A) Hyperkeratotic lesion on the right palm with significant rectification of the epidermis with orthohyperkeratosis; fibrosis and proliferation of small vessels are observed in the dermis (Hematoxylin & eosin ×200). (B) Hyperpigmentation on the abdomen with vacuolar degeneration foci of basal cells with mild pigmentary incontinence (Hematoxylin & eosin ×400). (C) Fontana-Masson staining demonstrating irregular distribution of melanin in the epidermis and pigmentary incontinence (×200).

Her only son had bilateral palmoplantar hyperkeratosis, micronychia, and leukoplakia on the tongue. He underwent a bone marrow transplant due to bone marrow aplasia at the age of four, causing significant thrombocytopenia. DNA sequencing revealed a mutation in the *TINF2* gene – p.Arg282His (c.845 G>A), described in autosomal dominant cases.

Telomeres consist of identical repeats of amino acids, which have a protective effect on chromosomes and shorten throughout life. Mutations in the proteins involved in this process lead to premature cell aging.^1^, ^2^

Dyskeratosis congenita is a rare multisystem telomeropathy.^3^, ^4^ It has characteristic mucocutaneous manifestations: reticulated hyperpigmentation of the skin, dystrophic nails, both present in the patient's case, and leukokeratosis in the mucous membranes,^3^, ^4^ identified only in the patient's son.

This patient's segmental clinical presentation is likely due to mosaicism, a later mutation, called a postzygotic mutation, resulting in two cell populations, either carrying the mutation or not, with the clinical repercussions of changes following embryonic lines, seen here in the nail involvement and pigmentary alteration. This phenomenon also occurs in the gonads, where two populations of cells are present, known as gonadal mosaicism. Fertilization of an egg carrying the mutation leads to a more severe condition, as seen in the patient's son, with all of his nails affected, leukoplakia, and bone marrow aplasia, interestingly without pigmentation changes.

The nails are most commonly affected and may disappear or be absent at birth, leading to a tapered appearance of the fingers. The variety of changes seen in this patient makes diagnosis difficult. Anonychia, onychoschizia, longitudinal striae or fractures, trachyonychia, koilonychia, pterygium, leukodynia, and melanonychia have already been described.^1^, ^2^

The mutation found has been previously described,^4^, ^5^, ^6^ affecting not telomerase, but a complex called the telosome, which protects telomeres and regulates telomerase,^7^ leading to premature telomere shortening.^5^, ^6^

In a cohort of 175 patients with DC,^5^ *TINF2* gene involvement was demonstrated in 33 (18.8%, slightly higher than other reports)^1^ of whom; 32 had aplastic anemia. As control, they used 244 patients with bone marrow failure, and three individuals had a mutation in this gene, exemplifying the complexity of the issue.^5^ Mutations in *TINF2* can lead to retinopathy, also called Revesz syndrome.^8^

Men are more affected than women,^1^ which highlights the importance of the X-linked recessive inheritance pattern. Women with X-linked DC tend to have milder phenotypes and less severe clinical presentations. This is due to the phenomenon of lyonization,^7^ a process that inactivates one of the two X chromosomes in female cells, generating reduced transcriptional activity. In these cases, symptoms always appear at an older age.^7^

Delayed diagnosis of DC is due to the broad spectrum of clinical presentations, the lack of specific laboratory tests, and the absence of conclusive histopathology,^3^, ^4^ as seen in the present case. Dermatologists should be alert, as the disease can present with serious hematological complications and malignancies, such as leukemia, squamous cell carcinoma from leukokeratosis, and non-Hodgkin's lymphoma.

## ORCID ID

Cecília Mazzoleni Facchini: 0009-0001-0266-2185

Camila Urach Colpo: 0009-0009-6512-6804

Manuela Sallaberry Maciel: 0009-0003-1052-8415

Ana Júlia Baschirotto Custódio: 0009-0001-3404-8323

Aline Paganelli: 0009-0005-8837-7332

## Financial support

None declared.

## Authors’ contributions

Hiram Larangeira de Almeida Jr.: Approval of the final version of the manuscript; design and planning of the study; drafting and editing of the manuscript; collection, analysis, and interpretation of data; effective participation in research orientation; intellectual participation in the propaedeutic and/or therapeutic conduct of the studied cases; critical review of the literature; critical review of the manuscript.

Cecília Mazzoleni Facchini: Approval of the final version of the manuscript; design and planning of the study; drafting and editing of the manuscript; collection, analysis, and interpretation of data; intellectual participation in the propaedeutic and/or therapeutic conduct of the studied cases; critical review of the literature; critical review of the manuscript.

Camila Urach Colpo: Approval of the final version of the manuscript; design and planning of the study; drafting and editing of the manuscript; collection, analysis, and interpretation of data; intellectual participation in the propaedeutic and/or therapeutic conduct of the studied cases; critical review of the literature; critical review of the manuscript.

Manuela Sallaberry Maciel: Approval of the final version of the manuscript; design and planning of the study; drafting and editing of the manuscript; collection, analysis, and interpretation of data; critical review of the literature; critical review of the manuscript.

Ana Júlia Baschirotto Custódio: Approval of the final version of the manuscript; design and planning of the study; drafting and editing of the manuscript; collection, analysis, and interpretation of data; critical review of the literature; critical review of the manuscript.

Aline Paganelli: Approval of the final version of the manuscript; design and planning of the study; drafting and editing of the manuscript; collection, analysis, and interpretation of data; critical review of the literature; critical review of the manuscript.

## Availability of research data

Not applicable.

## Conflicts of interest

None declared.
